# Paediatric safety signals identified in VigiBase: Methods and results from Uppsala Monitoring Centre

**DOI:** 10.1002/pds.4734

**Published:** 2019-02-15

**Authors:** Kristina Star, Lovisa Sandberg, Tomas Bergvall, Imti Choonara, Pia Caduff‐Janosa, I. Ralph Edwards

**Affiliations:** ^1^ Research Section Uppsala Monitoring Centre Uppsala Sweden; ^2^ Department of Public Health and Caring Sciences Uppsala University Uppsala Sweden; ^3^ Division of Medical Sciences and Graduate Entry Medicine University of Nottingham Derby UK

**Keywords:** adverse drug reaction reporting systems, individual case safety reports, paediatrics, pharmacoepidemiology, pharmacovigilance

## Abstract

**Purpose:**

The purpose of this study is to uncover previously unrecognised risks of medicines in paediatric pharmacovigilance reports and thereby advance a safer use of medicines in paediatrics.

**Methods:**

Individual case safety reports (ICSRs) with ages less than 18 years were retrieved from VigiBase, the World Health Organization (WHO) global database of ICSRs, in September 2014. The reports were grouped according to the following age spans: 0 to 27 days; 28 days to 23 months; 2 to 11 years; and 12 to 17 years. vigiRank, a data‐driven predictive model for emerging safety signals, was used to prioritise the list of drug events by age groups. The list was manually assessed, and potential signals were identified to undergo in‐depth assessment to determine whether a signal should be communicated.

**Results:**

A total of 472 drug‐event pairs by paediatric age groups were the subject of an initial manual assessment. Twenty‐seven drug events from the two older age groups were classified as potential signals. An in‐depth assessment resulted in eight signals, of which one concerned harm in connection with off‐label use of dextromethorphan and another with accidental overdose of olanzapine by young children, and the remaining signals referred to potentially new causal associations for atomoxetine (two signals), temozolamide, deferasirox, levetiracetam, and desloratadine that could be relevant also for adults.

**Conclusions:**

Clinically relevant signals were uncovered in VigiBase by using vigiRank applied to paediatric age groups. Further refinement of the methodology is needed to identify signals in reports with ages under 2 years and to capture signals specific to the paediatric population as a risk group.

**KEY POINTS**

- Signal detection of global individual case safety reports for paediatric age groups uncovered previously unrecognised risks of medicines.
- Three signals were further evaluated and subsequently added to the product label, providing new information for patients, caregivers, and healthcare professionals to consider prior to and during therapy.

## 1 INTRODUCTION

To minimise harm from medication use, healthcare professionals and patients need to know about the risks. Postmarketing reporting systems provide opportunities to increase our knowledge of risks that were not recognised in the premarketing clinical trials. The knowledge gained will constitute the basis for prevention and mitigation of patient harm by manufacturers, regulatory authorities, and healthcare institutions.

To detect previously unrecognised rare adverse drug reactions (ADRs), a wide population coverage is required. Hence, the World Health Organization (WHO) Programme for International Drug Monitoring was established in 1968 to ensure that safety concerns are identified, shared, and acted upon. The currently over 130 full member countries (April 2018) can access VigiBase, the WHO global database of individual case safety reports (ICSRs), as a reference source for national investigations. The Uppsala Monitoring Centre (UMC)^1^ that maintains VigiBase complements these national efforts by conducting periodical open‐ended signal detection screenings of global data. A safety signal, in this context, is a hypothesis of “a new potentially causal association, or a new aspect of a known association, between an intervention and an event,”^2^, ^3^ and “open ended” refers to screening of data without a prior hypothesis.

To use the opportunities of the broad coverage in VigiBase, UMC has begun to screen for safety signals in subgroups, eg, a specific type of reporters such as patients,^4^ geographical areas, and drug groups. Disproportionality analyses (used to highlight statistical associations for further evaluation^5^, ^6^) in subgroups of reports have been shown to uncover previously unknown associations^7^ and even improve performance compared with using the complete data set of a postmarketing reporting system.^8^ The first subgroup to be the subject for signal detection screening at UMC was reports within the paediatric age group.

Information on the safety and efficacy of a medicine used for neonates, infants, children, and adolescents is limited if individuals with these ages were not included in the premarketing clinical trials. Drug toxicity is poorly reported in paediatric clinical trials,^9^, ^10^ particularly where clinical trials involve both adults and children.^11^ As a consequence, information on dose recommendations, precautions, warnings, and ADR profiles specific to paediatric age groups can be lacking when prescribing and administering medicines to these patients.^12^ Children experience a wide range of ADRs, as described from national pharmacovigilance databases,^13^ and the reporting pattern differs both from reports for adults and between paediatric age subgroups.^14^ In order to increase knowledge for the safer use of medicines in the paediatric population, VigiBase reports were screened to uncover previously unrecognised risks of medicines in this age group.

## 2 METHODS

### 2.1 Scope

Signal detection and assessment at UMC follow a three‐step process as described in Table 1. The adjustments made to capture signals in reports on paediatric ages are further described for each of these steps in this section.

**Table 1:** Signal detection process at Uppsala Monitoring Centre (UMC)

I	First‐pass statistical screening	Exclusion and inclusion criteria are applied to a designated data set, and data‐driven methodologies and filters are implemented to a list of drug‐event pairs (coupled with or without a subgroup) or drug‐drug events.
II	Initial manual assessment	UMC assessors manually review the list of drug‐event pairs to identify potential signals to undergo in‐depth assessment. The review includes the following: • checking of whether the event is already well‐described in the product information; and • a brief review of the individual reports to exclude report series that displays an obvious alternate and more likely explanation for the association or lacks sufficient information for assessment.
III	In‐depth manual assessment	UMC staff or external experienced scientists and clinicians conduct causality assessment of the individual case reports and review the literature on the topic to compile evidence for or against a signal.^15^ Then, a decision is made whether or not the strength of the report series supports the communication of a signal.

“Drug‐event pairs” refer to clusters of ICSRs (denoted “reports” in this paper) with the same suspect or interacting drug and the same event. The preferred base level (active ingredient) in the WHO Drug dictionary was used to classify the “drug,” and the preferred term from the WHO‐Adverse Reaction Terminology (WHO‐ART) was used to classify the “event” in the signal detection screening of reports in the paediatric age group.

If the in‐depth assessment suggests that a signal should be communicated, the hypothesis is presented with data and arguments^16^ in SIGNAL. If a patent holder of the medicinal product in question can be identified, they are given the opportunity to respond to the signal in the same edition of SIGNAL. The signals are distributed to members of the WHO Programme for International Drug Monitoring and subsequently published in the WHO Pharmaceuticals Newsletter.^17^

### 2.2 First‐pass statistical screening of paediatric reports

#### 2.2.1 Designated data set

**Figure 1:**
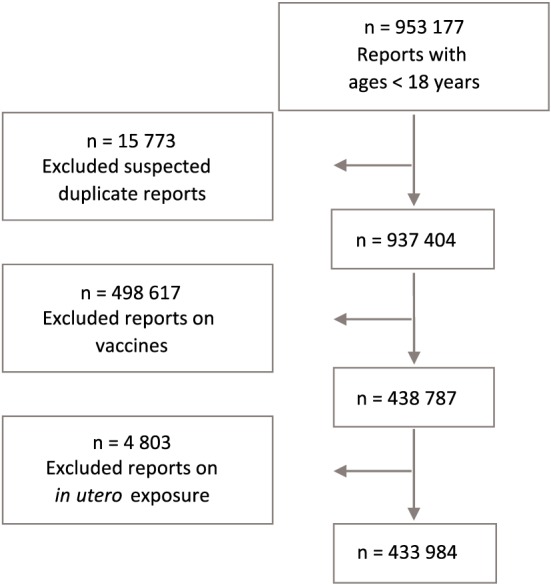
Number of reports included and excluded from the data set used in signal detection of paediatric age groups. The numbers in this flowchart have been reconstructed from the VigiBase database in 2017 by using the same cut‐off date as for the paediatric data set used during screening. However, because changes are continuously being made in the database, it was not possible to present exact figures as used in the paediatric signal detection screening in September 2014 [Colour figure can be viewed at wileyonlinelibrary.com]

The data set used for the first‐pass statistical screening of paediatric ages contained reports entered in VigiBase up to 1 September 2014 and was restricted to reports with ages less than 18 years. Figure 1 displays the number and type of reports that were included and excluded from the data set. Suspected duplicate reports were excluded by using the vigiMatch, an algorithm for automatic duplicate detection.^18^, ^19^ Reports on vaccines were excluded because vaccine reports were designated to a separate screening to allow for capturing age‐independent reports, hence not restricted to paediatric ages. Reports recording harm of newborns resulting from in utero exposure are not sufficiently captured in a data set restricted to paediatric ages, since these reports can be given with the mother's age or with no age specified; therefore, reports indicating in utero exposure were excluded and designated to a separate review.

Table 2 displays the exclusion criteria applied to drug‐event pairs within the complete paediatric age group (reports with ages less than 18 y), aiming to generate emerging, and global issues for review.

**Table 2:** Exclusion criteria for drug‐event pairs within the complete paediatric age group (reports with ages less than 18 y)

Exclusion Criteria for Drug‐Event Pairs With the Following:	Rationale
<3 or >30 reports	A restriction was made to drug‐event pairs to represent between three and 30 reports. The lower threshold was set to enable enough reports for assessment, and the limit to the maximum number of reports was set to increase the likelihood of capturing previously unknown problems (ie, signals) as well as rare adverse drug reactions, which holds the primary purpose of the international compilation of reports in VigiBase.
Single‐reported country	To complement national centres by focusing on global problems
No reports received in VigiBase after 1 January 2012	To be relevant and capture current problems

The reports were grouped according to four paediatric age ranges^20^: 0 to 27 days (neonates); 28 days to 23 months (infants); 2 to 11 years (children); 12 to 17 years (adolescents). Ages are defined in completed days, months, or years. We chose to acknowledge the vast differences between neonates and almost full‐grown adults and anticipated that the context of the reported event and potential confounders would be more obvious for the assessor when displaying data for each paediatric age group separately.

#### 2.2.2 Data‐driven screening method

**Figure 2:**
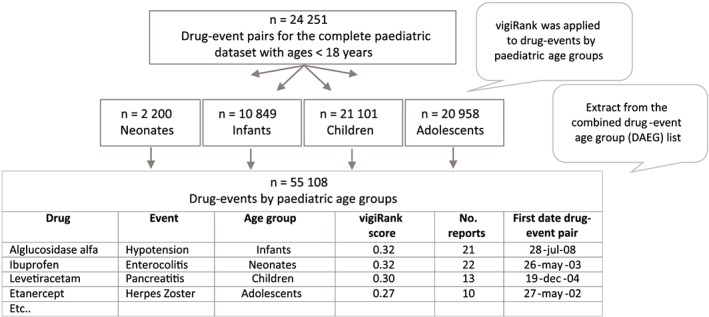
Extract from drug‐event age group (DEAG) list used during initial manual assessment. Twenty‐four thousand two hundred fifty one denotes unique drug‐event pairs for the complete paediatric data set with ages less than 18 years after applying exclusion criteria specified in Table 2 [Colour figure can be viewed at wileyonlinelibrary.com]

A screening list was generated representing drug‐event pairs reported within any of the four paediatric age groups. vigiRank, a data‐driven predictive model, was used to prioritise report series likely to be signals by weighing disproportionate reporting patterns, report completeness, recentness of reports, geographical spread, and the availability of report narratives.^21^ vigiRank scores were computed for the drug events within each of the four paediatric age groups. The drug events of the four age groups were thereafter combined into one drug‐event age group (DEAG) list, which was prioritised according to the vigiRank scores. The DEAGs could be represented by the same vigiRank scores, so a secondary sorting was applied by prioritising the report recentness of the DEAG. See extract from the listing in Figure 2.

#### 2.2.3 Filters applied to the drug‐event paediatric age‐group list

The DEAG list included 55 108 posts for review, so we applied and tested four different filters in the initial manual assessment as presented in Table 3. At previous screenings of VigiBase, WHO‐ART critical terms (considered as being indicative of seriousness) had been used to prioritise report series for review.^22^ We were uncertain whether serious problems specific to paediatric ages were captured with critical terms. Diarrhoea, which can be a life‐threatening event for an infant, was, for example, not designated to be a critical term. Therefore, we identified report series referring to serious events using the International Conference of Harmonisation (ICH) seriousness criteria,^23^ flagging DAEGs where all reports in E2B format fulfilled one of the ICH seriousness criteria.

**Table 3:** Filters applied to the drug event paediatric age group list during initial manual assessment

Filter	Rationale
Serious event^a^ and new drug in the age group^b^ (≥2009)	To capture problems that were unlikely to be discovered in clinical trials or in national databases. A “new drug” reported for the age group can suggest that a new product has been approved for the age group or that a new clinical use of the drug is emerging in the age group and therefore needs monitoring.
Serious event^a^ and new drug in the age group^b^ (≥2005)	
Serious event^a^ (no restriction to the newness of the drug)	Because this was the first screening of paediatric global data, the test included drug events that represented a wider scope, to allow previously unrecognised safety issues to emerge also for drugs, which had been on the market for a long time.
Negative disproportionality measure in the full VigiBase data set^c^ and automatic exclusion of labelled adverse drug reactions based on the US Food and Drug Administration (FDA) product label^24^ and the European Summary of Product Characteristics (EU SmPC).^25^	To increase the chances of capturing signals specific to the paediatric age group but still being unknown and less likely to have been highlighted in previous signal detection screenings of the full VigiBase data set.

a Reports series referring to serious events using the ICH seriousness criteria,^23^ flagging pairs where all reports in the E2B format fulfilled one of the ICH seriousness criteria.^23^

b New drugs were defined as drugs first reported to VigiBase in the specific paediatric age group on/after 1st of January 2009 (≥ 2009) or on/after 1st of January 2005 (≥ 2005).

c The negative disproportionality measure referred here is based on the negative lower end point of the 95% credibility interval of the Information Component^5^, ^6^ (IC025 < 0) and denotes less reporting than expected in the full VigiBase data set.

### 2.3 Initial manual assessment

The aim of the initial manual assessment was to identify potential signals that should proceed to in‐depth assessment. A multidisciplinary team of pharmacists, nurses, data scientists, and physicians (including a paediatrician/clinical pharmacologist) manually assessed the DEAG list. The assessors could select all paediatric age groups with the same drug event in the same assessment and thereby occasionally deviate from the prioritisation by vigiRank. The product information was scrutinised to determine whether the drug‐event pair should be considered “known” for the age group. If the event was labelled but the drug was not approved for the specific age group, additional sources, used by paediatricians, were reviewed to check whether these sources had listed the event and therefore could be considered to be known. For safety labelling and approval status, the UK Summary of Product Characteristics^26^ and product labels of drugs approved by the US Food and Drug Administration (FDA)^27^ were consulted. The British National Formulary for Children^28^ or NeoFax and Paediatrics^29^ were referenced to represent sources used by paediatricians. Whenever the drug could not be found in the aforementioned sources, DrugDex,^30^ Martindale,^31^ or other national product information was reviewed.

**Figure 3:**
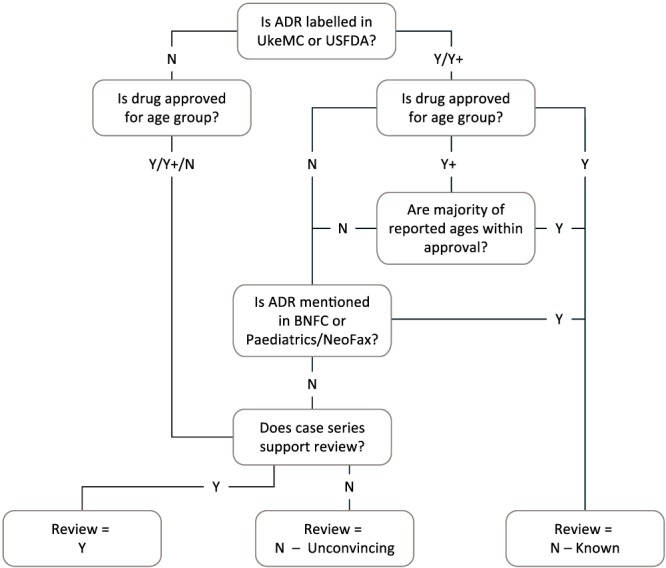
Decision tree for classification of drug‐event pairs by paediatric age groups during initial manual assessment [Colour figure can be viewed at wileyonlinelibrary.com]

Each post in the DEAG list was categorised according to a decision tree (Figure 3) and recorded with any of the following outcomes:

- Potential signal, needing further in‐depth manual assessment
- Known, considered well‐described for the specific age group in the product information
- Nonsignal, report series suggests alternative more likely explanations for the event, such as coreported drugs, or lacks sufficient/relevant data for assessment
- Keep under review (KUR), needing time to gather more/better documented reports

During the assessment, a system for graphical overview and access to the global individual reports was used in addition to statistics displayed for the DEAGs via a separate software interface with information such as the following:

- selected information tabulated for each individual report, including narratives (when available)
- summary statistics for the report series such as
  - number of reports
  - age range
  - dates for when the first report on the drug and for the drug event was entered into VigiBase in the specific age group
  - number of reports with fatal outcome, any seriousness criteria, positive dechallenge and rechallenge, and reports with sole suspected drugs
  - statistics constituting the basis for the vigiRank score
  - disproportionality measures for the age group and the full data set

The assessors spent 1 week on the task with the aim to identify 10 to 15 potential signals. The predefined number of potential signals was based on the estimated manual capacity required for the subsequent in‐depth assessment.

### 2.4 In‐depth manual assessment

Clinical experts from the UMC or external expert volunteers, who are part of the UMC Signal review panel, assessed the individual reports (representing the potential signals) in depth and reviewed the literature to compile evidence for or against a signal.^15^ The assessors could choose to restrict their assessment to reports on paediatric ages or include reports for other ages as well.

## 3 RESULTS

A total of 55 108 DEAGs were retrieved after applying the inclusion and exclusion criteria described in Figure 1 and Table 2. The median number of reports representing the DEAG posts was three reports, and 45% contained one to two reports (83% and 69% for the neonates and infants and 36% and 37% for the children and adolescents, respectively).

During the initial manual assessment, 472 DEAG posts (392 unique drug‐event pairs) were reviewed (neonates = 13; infants = 37; children = 217; adolescents = 205). The number and proportion of potential signals, as well as known ADRs for the age group/nonsignals/KURs, are displayed in Table 4. A total of 27 DEAGs and 21 unique drug‐event pairs were classified as potential signals requiring in‐depth manual assessment. The potential signals were all identified in the two older paediatric age groups.

**Table 4:** Results from the initial manual assessment: Number of assessed and categorised drug‐event pairs by paediatric age group

Assessed Drug‐Event Report Series	Neonates (n = 13)		Infants (n = 37)		Children (n = 217)		Adolescents (n = 205)		All Paediatric Ages (n = 472)	
	No.	%	No.	%	No.	%	No.	%	No.	%
Known^a^	2	15	11	30	131	60	147	72	291	62
Nonsignal^b^	11	85	26	70	68	31	47	23	152	32
Keep under review^c^	0	‐	0	‐	1	0.5	1	0.5	2	0.4
Potential signal^d^	0	‐	0	‐	17	7.8	10	4.9	27	5.7

a Considered well‐described for the specific age group in the product information.

b Reports suggested alternative for more likely explanations for the event, such as coreported drugs, or lacked sufficient/relevant data for assessment.

c Indicated potential signal but required time to gather more/better documented reports.

d Required further in‐depth manual assessment.

The filters applied to the DEAG list and that generated the greatest rate of potential signals were DEAG posts with a negative disproportional pattern in the full VigiBase data set and with labelled reactions excluded and serious events with new drugs (≥2009). A total of 10% and 7% of the DEAGs, respectively, were classified as potential signals when these filters had been applied in comparison with approximately 4.5% to 5% for the other filters, see Table 5.

**Table 5:** Number of drug events per paediatric age groups presented by each filter applied to the screening list during the initial manual assessment

Screening Filters	No. Drug‐Event Pairs by Paediatric Age Groups	
	Total Assessed	Potential Signals
Serious events^a^ and new drugs in age group later than or equal to 2009^b^	82	6
Serious events^a^ (no restriction to drug)	377	19
Serious events^a^ and new drugs in age group later than or equal to 2005^b^	221	10
Negative disproportionality measure overall^c^ and not labelled^d^	126	13

The same drug‐event pairs (coupled with any or several of the four paediatric age groups) can be accounted for in more than one screening filter.

a Reports series referring to serious events using the ICH seriousness criteria,^23^ flagging pairs where all reports in the E2B format fulfilled one of the ICH seriousness criteria.

b New drugs were defined as drugs first reported to VigiBase in the specific paediatric age group on/after 1st of January 2009 or on/after 1st of January 2005.

c The negative disproportionality measure referred here was based on the negative lower end point of the 95% credibility interval of the Information Component (IC025 < 0)^5^, ^6^ and denoted less reporting than expected in the full VigiBase data set.

d Automatic exclusion of labelled adverse drug reactions based on the US Food and Drug Administration (FDA) product label^24^ and European Summary of Product Characteristics (EU SmPC).^25^

The in‐depth assessment resulted in eight signals (2.0%, 8/392 unique drug‐event pairs), which were communicated within the WHO Programme via SIGNAL and published in the WHO Pharmaceuticals Newsletter in numbers 4 to 6 in 2015,^32^, ^33^, ^34^, ^35^, ^36^, ^37^ number 2 in 2016,^38^ and number 4 in 2017.^39^ A summary of each signal is displayed in Table 6. In all but the two patient safety signals concerning off‐label use and accidental overdose, the drug‐event report series was listed with a negative disproportional pattern in the full VigiBase data set.

**Table 6:** Summary of paediatric safety signals identified in VigiBase

Drug‐event SIGNAL Issue/WHO Pharmaceuticals Newsletter	Indication of Use	Highlighted in Screening: Observed^a^ vs Expected Number of Reports^6^/Age Group/Disproportionality Measure in Age‐ Independent Data Set^b^	In‐Depth Assessment Covered: Number of Reports/Age Range/No. Countries Represented	Additional Signal Features
Atomoxetine and dystonia^32^ April 2015/2015 no. 4	Attention deficit hyperactivity disorder	20 vs 31.9 reports/2‐11 y/negative	31 reports/5‐17 y/10 countries	• Thirteen cases recovered after drug withdrawal (based on available records). • In seven cases, antipsychotic medicines were cosuspected and were the likely cause for the dystonia. • Jaw‐closing dystonia with methylphenidate and dexamphetamine was signalled by The Netherlands Pharmacovigilance Centre Lareb^47^ in 2012.
Atomoxetine and neutropenia^33^ April 2015/2015 no. 6	Attention deficit hyperactivity disorder	14 vs 40.2 and 15 vs 37.8 reports with granulocytopenia/2‐11 and 12‐17 y/negative	25 reports/6‐17 y/seven countries	• Signal covered high level WHO‐ART term neutropenia. • Twenty reports with atomoxetine as the sole suspected drug. • Time to onset (eleven cases) ranged from 14 days to 10 months. • Seven cases recovered after drug withdrawal (based on available records).
Dextromethorphan and serious neurological disorders^36^ April 2015/2015 no. 5	Used in many cough, cold, and flu products approved from age 4 and 6 years in the United States and the United Kingdom, respectively	15 vs 1.6 reports with ataxia/2‐11 y/positive	110 reports with neurological disorders for ages less than 6 y	• Continuous to be reported also after 2008/2009 when a recommendation that these ages should not be given this medicine was issued. • Serious reactions such as ataxia, convulsions, dyskinesia, and coma had been reported. •Majority of reports were not reported with accidental intake of drug or overdose. • Better warnings for parents suggested.
Olanzapine and accidental drug intake by children^37^ April 2015/2015 no. 5	For schizophrenia and bipolar disorder in adults	11 vs 0.2 reports with miosis (majority coreported with accidental exposure)/2‐11 y/positive	20 reports with medication‐error‐related problems (eg, accidental exposure)/below the age of 6 y	• Reminder to consider the risk of accidental exposure to young children when prescribing these drugs to patients with potential decreased risk awareness. • Packaging is crucial to protect children from accidental exposure to these very potent drugs.
Temozolomide and oesophagitis^35^ April 2015/2015 no. 5	Malignant glioma	4 vs 0.06 reports/2‐11 y/negative	Nine reports/ages 8, 10, 62, 67, 69 y and four with unspecified age	•Three reports with temozolomide as the sole suspected drug. • Coreported with other drugs and terms. • Plausible pharmacological mechanism. • Oesophagitis plausible when considering other labelled events such as stomatitis, dysphagia, and gastroenteritis.
Deferasirox and pancreatitis^34^ June 2015/2015 no. 6	Chronic iron overload because of frequent blood transfusions in patients with beta thalassaemia major	9 vs 2.7 reports/12‐17 y/negative	14 reports/4‐16 y/five countries (any ages: 62 reports)	• Eleven reports with deferasirox as the sole suspected drug. • Time to onset (nine cases): 17 days to 5 years with a median of 11 months. • Six cases recovered after drug withdrawal (based on available records). • Four isolated cases from published clinical trials (two concerned elevated amylase levels). • Company responds by adding pancreatitis to the label.^42^
Levetiracetam and impaired renal function^38^ June 2015/2016 no. 2	Epilepsy	13 vs 2.9 reports with acute renal failure/12‐17 y/negative	14 paediatric reports/6 wk‐17 y/five countries/twelve well‐documented reports for adults assessed/more than 100 reports with any ages	• Signal covered reports with acute renal failure, azotaemia, renal function abnormal, and/or interstitial nephritis. • Several published case reports had been described for paediatric ages and adults. • Three of twelve well‐documented reports for adults described increasing creatinine levels after starting levetiracetam followed by a reduction to normal after drug withdrawal. • Acute kidney injury added to the label.^40^
Desloratadine, loratadine and weight increase^39^ April 2017/2017 no. 4	Allergic rhinitis and urticaria	8 vs 1.4 reports with desloratadine and weight increase/2‐11 y/negative	Any ages: 44 reports with desloratadine, of which eleven cases aged 4‐11 y/ 115 reports with loratadine, of which eleven cases aged 4‐10 y	• Assessment covered reports with desloratadine and loratadine with any age, with a focus on ages less than 12 years (paediatric cases in this signal). • Reports covered weight increase, obesity, and appetite increased. • Paediatric cases with desloratadine: five reports with sole suspect drug; two cases with a positive dechallenge and rechallenge. • Paediatric cases with loratadine: nine reports with sole suspected drug; five cases recovered after drug withdrawal, of which one was reported with a positive rechallenge. • Desloratadine and increased appetite were signalled by The Netherlands Pharmacovigilance Centre Lareb^48^ in 2011. • Weight increased^41^, ^49^ and appetite increased^41^ added to the label.

Abbreviations: WHO, World Health Organization; WHO‐ART, WHO‐Adverse Reaction Terminology. Full texts are found in the WHO Pharmaceuticals Newsletter.

a Observed = number of reports represented in the report series.

b The positive/negative disproportionality measure referred here was based on the positive/negative lower end point of the 95% credibility interval of the Information Component^5^, ^6^ and denoted more/less reporting than expected in the full VigiBase data set.

## 4 DISCUSSION

Clinically relevant signals were identified by screening paediatric age groups in VigiBase. Of the eight signals,^32^, ^33^, ^34^, ^35^, ^36^, ^37^, ^38^, ^39^ two concerned harm in connection to off‐label use and accidental overdose of medicines by young children. Six signals referred to new potentially causal associations, of which labelling changes have subsequently been made for three.^40^, ^41^, ^42^

The signals suggesting new associations for atomoxetine, temozolamide, deferasirox, levetiracetam, and desloratadine were also relevant for adults. None of these signals had a positive disproportional reporting pattern in the full VigiBase data set and had not previously been reviewed when using the full data set and disproportionality analyses as the screening method. So, in addition to finding signals relevant for the paediatric population, the focus on paediatric age groups enabled issues to be uncovered that are important for any age group.

In two signals, young children were identified as a particular risk group. These signals demonstrated the need for further action to prevent parents from administering dextromethorphan to young children despite warnings raised by authorities and to initiate measures to prevent accidental intake of antipsychotic medicines by young children. Historically, signal detection and assessment at UMC have focused on finding previously unknown associations between events and drugs, ie, compiling evidence for or against a causal relationship. In the review based on the paediatric ages, the patient group and its context were in focus as a possible risk group in addition to exploring a causal relationship. This required a different mindset when assessing the report series. In setting up for the current signal detection review, much effort and time were put into defining the scope and adjusting current statistical screening methodology to the paediatric population. In retrospect, however, more effort should have been made on guiding the assessors to capture signals specific to paediatric ages as a particular risk group.

No signals originated from the two youngest age groups (0‐27 d and 28 d‐23 mo). The time spans of these age groups (1 and 23 mo, respectively) are much shorter than for the two older age groups (10 and 6 y, respectively). Hence, the younger age groups have fewer reports overall, fewer drug‐event pairs, and fewer reports per drug‐event pair (83% and 69% of the pairs in these age groups were based on only one to two reports), resulting in lower vigiRank scores. Consequently, the vigiRank scores for the older age groups were competing with the scores for the younger age groups when the drug‐event pairs for the paediatric age groups were combined into the DAEG list. To better account for the younger ages in the future, these reports could be combined into one age group (<2 y), and/or less granular medical terms could be used to increase the number of reports per drug‐event pair. Alternative solutions could be to review drug events in these age groups separately to avoid a dominance of drug events from the older age groups or randomise an equal number of drug‐event pairs from each age group to undergo assessment.

Drug events by the four paediatric age groups were prioritised by vigiRank, and well‐described ADRs for the age groups were excluded from further evaluation. The proportion of identified signals based on the paediatric data in VigiBase was lower than a previous signal detection screening using the vigiRank on the full VigiBase data set^43^ (2.0% vs 3.1%). Known ADRs had a higher frequency in the paediatric data set (63% of all unique drug‐event pairs) compared with a previous screening of the full data set (41%),^43^ but when excluding the known ADRs from the denominator in the current and previous data sets, a similar proportion of identified signals was identified (5.5% vs 5.2%). The higher proportion of known ADRs for the paediatric drug events could be explained by that they represented large report series in the adult data set, hence increasing the likelihood of being known ADRs.

In the paediatric signal detection screening, we restricted the number of reports to 30 per drug‐event pair within the ages 0 to 17 years for reasons set out in Table 2. This restriction in the number of reports might have been too conservative, possibly resulting in missing important signals among paediatric reports. In future screenings, this limit to the number of reports should be reconsidered.

New initiatives are encouraged within paediatric pharmacovigilance. The European Medicines Agency (EMA) recently developed a statistical query that can be applied to EudraVigilance to highlight imbalances in reported drug‐event pairs for children as compared with adults. The query has been used to support EMA Scientific Committees in their investigation of specific paediatric safety concerns.^44^ The current description of signal detection of paediatric age groups refers to safety concerns identified via large‐scale hypotheses generation of VigiBase data and presents how these findings were identified using the vigiRank.

It should be noted that the signals presented in this paper are preliminary in nature and their status can change over time when more data on the problems identified are available. Also, the basis for the signals is a global pharmacovigilance reporting system, which has known limitations, such as the information being from a variety of sources, and the likelihood that the suspected adverse reaction being drug related is not the same in all cases.^45^

Spontaneous reporting systems have known strengths and limitations.^46^ Underreporting causes signals to be missed because ADRs are not always recognised or reported by caregivers, patients, or health professionals. Another weakness of the system is the possibility of various reporting biases, for example, unexpected overreporting because of media attention or because the medicine is undergoing intensive monitoring, which can influence quantitative analyses. Also, the in‐depth assessment of individual reports can be restricted because of poor quality data or lack of data. However, the method used for identifying drug‐event pairs for further evaluation at UMC, vigiRank, prioritises not only disproportionate reporting patterns but also informative report series and thereby increases the chances of a conclusive case assessment.^21^, ^43^

## 5 CONCLUSIONS

Clinically relevant signals were uncovered in VigiBase by using the vigiRank applied to paediatric age groups. Three of these safety concerns were subsequently added to the product label, providing new information for patients, caregivers, and healthcare professionals to consider prior to and during therapy. Further refinement of the methodology is needed to identify signals in the youngest paediatric age groups and to capture signals specific to the paediatric population as a risk group.

## ETHICS STATEMENT

The authors state that no ethical approval was needed.

## CONFLICT OF INTEREST

The authors declare no conflict of interest.

## References

[1] Uppsala Monitoring Centre. Available from: https://www.who‐umc.org/. Accessed Apr 27, 2018.

[2] Hauben M , Aronson JK . Defining 'signal' and its subtypes in pharmacovigilance based on a systematic review of previous definitions. Drug Saf. 2009;32(2):99‐110. 19236117 10.2165/00002018-200932020-00003

[3] Council for International Organisations of Medical Sciences (CIOMS) Working Group VIII . Practical aspects of signal detection in pharmacovigilance CIOMS; 2010.

[4] Watson S , Chandler RE , Taavola H , et al. Safety concerns reported by patients identified in a collaborative signal detection workshop using VigiBase: results and reflections from Lareb and Uppsala Monitoring Centre. Drug Saf. 2018;41(2):203‐212. 28933055 10.1007/s40264-017-0594-2 PMC5808049

[5] Bate A , Lindquist M , Edwards IR , et al. A Bayesian neural network method for adverse drug reaction signal generation. Eur J Clin Pharmacol. 1998;54(4):315‐321. 9696956 10.1007/s002280050466

[6] Norén GN , Hopstadius J , Bate A . Shrinkage observed‐to‐expected ratios for robust and transparent large‐scale pattern discovery. Stat Methods Med Res. 2013;22(1):57‐69. 21705438 10.1177/0962280211403604 PMC6331976

[7] Hopstadius J, Norén GN Robust discovery of local patterns: subsets and stratification in adverse drug reaction surveillance. Proceedings of the 2nd ACM SIGHIT International Health Informatics Symposium, Miami, FL, 2012; 265‐274.

[8] Seabroke S , Candore G , Juhlin K , et al. Performance of stratified and subgrouped disproportionality analyses in spontaneous databases. Drug Saf. 2016;39(4):355‐364. 26748507 10.1007/s40264-015-0388-3

[9] de Vries TW , van Roon EN . Low quality of reporting adverse drug reactions in paediatric randomised controlled trials. Arch Dis Child. 2010;95(12):1023‐1026. 20551194 10.1136/adc.2009.175562

[10] Nor Aripin KN , Choonara I , Sammons HM . Systematic review of safety in paediatric drug trials published in 2007. Eur J Clin Pharmacol. 2012;68(2):189‐194. 21858432 10.1007/s00228-011-1112-6 PMC3256313

[11] Anderson M , Choonara I . A systematic review of safety monitoring and drug toxicity in published randomised controlled trials of antiepileptic drugs in children over a 10‐year period. Arch Dis Child. 2010;95(9):731‐738. 20522477 10.1136/adc.2009.165902

[12] Star K , Edwards IR . Pharmacovigilance for children's sake. Drug Saf. 2014;37(2):91‐98. 24446277 10.1007/s40264-013-0133-8

[13] Cliff‐Eribo KO , Sammons H , Choonara I . Systematic review of paediatric studies of adverse drug reactions from pharmacovigilance databases. Expert Opin Drug Saf. 2016;15(10):1321‐1328. 27501085 10.1080/14740338.2016.1221921

[14] Star K , Noren GN , Nordin K , Edwards IR . Suspected adverse drug reactions reported for children worldwide: an exploratory study using VigiBase. Drug Saf. 2011;34(5):415‐428. 21513364 10.2165/11587540-000000000-00000

[15] Hill AB . The environment and disease: association or causation? Proc R Soc Med. 1965;58:295‐300. 14283879 10.1177/003591576505800503 PMC1898525

[16] Meyboom RH , Egberts AC , Edwards IR , Hekster YA , de Koning FH , Gribnau FW . Principles of signal detection in pharmacovigilance. Drug Saf. 1997;16(6):355‐365. 9241490 10.2165/00002018-199716060-00002

[17] World Health Organization . WHO Pharmaceuticals Newsletter. Available from: http://www.who.int/medicines/publications/newsletter/en/. Accessed Nov 10, 2017.

[18] Tregunno PM , Fink DB , Fernandez‐Fernandez C , Lazaro‐Bengoa E , Noren GN . Performance of probabilistic method to detect duplicate individual case safety reports. Drug Saf. 2014;37(4):249‐258. 24627310 10.1007/s40264-014-0146-y

[19] Norén GN , Orre R , Bate A , Edwards IR . Duplicate detection in adverse drug reaction surveillance. Data Min Knowl Disc. 2007;14(3):305‐328.

[20] International Conference on Harmonisation of technical requirements for registration of pharmaceuticals for human use. ICH harmonised tripartite guideline. Clinical Investigation of Medicinal Products in the Pediatric Population E11. Available from: http://www.ich.org/fileadmin/Public_Web_Site/ICH_Products/Guidelines/Efficacy/E11/Step4/E11_Guideline.pdf. Accessed Sep 10, 2018.

[21] Caster O , Juhlin K , Watson S , Noren GN . Improved statistical signal detection in pharmacovigilance by combining multiple strength‐of‐evidence aspects in vigiRank. Drug Saf. 2014;37(8):617‐628. 25052742 10.1007/s40264-014-0204-5 PMC4134478

[22] Ståhl M , Lindquist M , Edwards IR , Brown EG . Introducing triage logic as a new strategy for the detection of signals in the WHO drug monitoring database. Pharmacoepidemiol Drug Saf. 2004;13(6):355‐363. 15170764 10.1002/pds.894

[23] International Conference on Harmonisation of Technical Requirements for Registration of Pharmaceuticals for Human Use . ICH Harmonised tripartite guideline. Post‐approval safety data management: definitions and standards for expedited reporting E2D. 2003.

[24] National Library of Medicine . DailyMed [Internet]. 2014 Available from: https://dailymed.nlm.nih.gov/dailymed/index.cfm.

[25] European Medicines Agency . Adverse drug reactions database [Internet]. 2014 Available from: http://www.imi‐protect.eu/adverseDrugReactions.shtml.

[26] Electronic Medicines Compendium . Summary of product characteristics [Internet]. 2014 Available from: https://www.medicines.org.uk/emc/.

[27] US Food and Drug Administration . Drugs@FDA: FDA approved drug products [Internet]. 2014 Available from: https://www.accessdata.fda.gov/scripts/cder/daf/.

[28] Paediatric Formulary Committee . British National Formulary (BNF) for Children. London: Published jointly by BMJ Publishing Group Ltd, Royal Pharmaceutical Society and Royal College of Paediatrics and Child Health Publications Ltd.; 2014.

[29] NeoFax and Pediatrics [Internet]. Micromedex Solutions. Truven Health Analytics, Inc. Ann Arbor, MI. 2014 Available from: http://www.micromedexsolutions.com.

[30] DrugDex [Internet]. Micromedex Solutions. Truven Health Analytics, Inc. Ann Arbor, MI. 2014 Available from: http://www.micromedexsolutions.com.

[31] Martindale [Internet]. Micromedex Solutions. Truven Health Analytics, Inc. Ann Arbor, MI. 2014 Available from: http://www.micromedexsolutions.com.

[32] Boyd I. Atomoxetine and dystonia in paediatric patients. *WHO Pharmaceuticals Newsletter* [Internet]. 2015(No.4):20–23. Available from: http://apps.who.int/iris/bitstream/10665/255499/1/WPN‐2015‐04‐eng.pdf?ua=1.

[33] Boyd I. Atomoxetine and neutropenia in paediatric patients. *WHO Pharmaceuticals Newsletter* [Internet]. 2015(No.6):16–19. Available from: http://www.who.int/medicines/publications/pharmnewsletter6‐2015.pdf

[34] Boyd I. Deferasirox and pancreatitis in paediatric patients. *WHO Pharmaceuticals Newsletter* [Internet]. 2015(No.6):20–23. Available from: http://www.who.int/medicines/publications/pharmnewsletter6‐2015.pdf

[35] Carvajal A. Temozolomide and oesophagitis. *WHO Pharmaceuticals Newsletter* [Internet]. 2015(No.5):19–22. Available from: http://apps.who.int/iris/bitstream/10665/255497/1/WPN‐2015‐05‐eng.pdf?ua=1.

[36] Sandberg L , Watson S . Dextromethorphan and serious neurological disorders in children. *WHO Pharmaceuticals Newsletter* [Internet]. 2015(No.5):18. Available from: http://apps.who.int/iris/bitstream/10665/255497/1/WPN‐2015‐05‐eng.pdf?ua=1.

[37] Sandberg L , Watson S . Olanzapine and accidental drug intake by children. *WHO Pharmaceuticals Newsletter* [Internet]. 2015(No.5):19. Available from: http://apps.who.int/iris/bitstream/10665/255497/1/WPN‐2015‐05‐eng.pdf?ua=1.

[38] Choonara I , Star K . Levetiracetam and impaired renal function. *WHO Pharmaceuticals Newsletter* [Internet]. 2016(No.2):18–23. Available from: http://apps.who.int/iris/bitstream/10665/255494/1/WPN‐2016‐02‐eng.pdf?ua=1.

[39] Viola E , Conforti A . Desloratadine, loratadine and weight increase in children. *WHO Pharmaceuticals Newsletter* [Internet]. 2017(No.4):15–19. Available from: http://apps.who.int/iris/bitstream/10665/258800/1/WPN‐2017‐04‐eng.pdf.

[40] Electronic Medicines Compendium . Summary of product characteristics for Keppra 500 mg film‐coated tablets. Updated on 2 Jan 2017 Available from: https://www.medicines.org.uk/emc/product/2293/smpc/history. Accessed Nov 11, 2017.

[41] Electronic Medicines Compendium . Summary of product characteristics for Neoclarityn 5 mg film‐coated tablets. Updated on 21 Dec 2017 Available from: https://www.medicines.org.uk/emc/product/1639/smpc/history. Accessed Apr 15, 2018.

[42] Novartis . Deferasirox and pancreatitis in paediatric patients. Response from Novartis. *WHO Pharmaceuticals Newsletter* [Internet]. 2015(No.6):23–25. Available from: http://www.who.int/medicines/publications/pharmnewsletter6‐2015.pdf

[43] Caster O , Sandberg L , Bergvall T , Watson S , Noren GN . vigiRank for statistical signal detection in pharmacovigilance: first results from prospective real‐world use. Pharmacoepidemiol Drug Saf. 2017;26(8):1006‐1010. 28653790 10.1002/pds.4247 PMC5575476

[44] Blake KV , Saint‐Raymond A , Zaccaria C , Domergue F , Pelle B , Slattery J . Enhanced paediatric pharmacovigilance at the European Medicines Agency: a novel query applied to adverse drug reaction reports. Paediatr Drugs. 2016;18(1):55‐63. 26597489 10.1007/s40272-015-0154-0

[45] Uppsala Monitoring Centre. WHO Collaborating Centre for International Drug Monitoring . Caveat document. Available from: https://www.who‐umc.org/media/1417/umc_caveat_201605.pdf. Accessed Feb 10, 2018.

[46] Goldman SA . Limitations and strengths of spontaneous reports data. Clin Ther. 1998;20(Suppl C):C40‐C44. 9915089 10.1016/s0149-2918(98)80007-6

[47] The Netherlands Pharmacovigilance Centre Lareb . Methylphenidate, dexamphetamine and trismus. Available from: https://databankws.lareb.nl/Downloads/KWB_2012_4_methyl1.pdf. Accessed Sept 17, 2018.

[48] The Netherlands Pharmacovigilance Centre Lareb . Desloratadine and increased appetite. Available from: https://databankws.lareb.nl/Downloads/kwb_2011_3_deslo.pdf. Accessed Sept 17, 2018.

[49] Electronic Medicines Compendium . Summary of product characteristics for Clarityn allergy 10mg tablets. Updated on 15 Feb 2018. Available from: https://www.medicines.org.uk/emc/product/3505/smpc/history. Accessed Apr 15, 2018.

